# Telomere length, oxidative and epigenetic changes in blood DNA of patients with exacerbated psoriasis vulgaris^☆^

**DOI:** 10.1016/j.abd.2022.01.008

**Published:** 2022-10-29

**Authors:** Martin Beranek, Pavel Borsky, Zdenek Fiala, Ctirad Andrys, Kvetoslava Hamakova, Marcela Chmelarova, Helena Kovarikova, Adam Karas, Jan Kremlacek, Vladimir Palicka, Lenka Borska

**Affiliations:** a Institute of Clinical Biochemistry and Diagnostics, University Hospital Hradec Kralove and Faculty of Medicine, Hradec Kralove, Charles University, Czech Republic; b Department of Biochemical Sciences, Faculty of Pharmacy, Hradec Kralove, Charles University, Czech Republic; c Institute of Preventive Medicine, Charles University, Faculty of Medicine, Hradec Kralove, Czech Republic; d Institute of Clinical Immunology and Allergology, Charles University, Faculty of Medicine, Hradec Kralove, Czech Republic; e Clinic of Dermatology and Venereology, University Hospital Hradec Kralove, Czech Republic; f Department of Medical Biophysics, Charles University, Faculty of Medicine, Hradec Kralove, Czech Republic

**Keywords:** Blood, DNA, Epigenomics, Methylation, Oxidative stress, Psoriasis, Telomere

## Abstract

**Background:**

The pathogenesis of psoriasis vulgaris involves changes in DNA molecules, genomic instability, telomere attrition, and epigenetic alterations among them. These changes are also considered important mechanisms of aging in cells and tissues.

**Objective:**

This study dealt with oxidation damage, telomere length and methylation status in DNA originating from peripheral blood of 41 psoriatic patients and 30 healthy controls.

**Methods:**

Oxidative damage of serum DNA/RNA was determined immunochemically. Real-time PCR was used for the analysis of the telomere length. ELISA technique determined levels of 5-methylcytosine in blood cells' DNA.

**Results:**

Oxidative damage of serum DNA/RNA was higher in patients than in controls (median, 3758 vs. 2286 pg/mL, p < 0.001). A higher length of telomeres per chromosome was found in patients whole-cell DNA than in controls (3.57 vs. 3.04 kilobases, p = 0.011). A negative correlation of the length of telomeres with an age of the control subjects was revealed (Spearman’s rho = -0.420, p = 0.028). Insignificantly different levels of 5-methylcytosine in patients and controls were observed (33.20 vs. 23.35%, p = 0.234). No influences of sex, smoking, BMI, PASI score, and metabolic syndrome on the methylation status were found.

**Study limitations:**

i) A relatively small number of the participants, particularly for reliable subgroup analyses, ii) the Caucasian origin of the participants possibly influencing the results of the parameters determined, and iii) Telomerase activity was not directly measured in serum or blood cells.

**Conclusion:**

The study demonstrated increased levels of oxidized DNA/RNA molecules in the serum of patients with exacerbated psoriasis vulgaris. The results were minimally influenced by sex, the presence of metabolic syndrome, or cigarette smoking. In the psoriatic blood cells' DNA, the authors observed longer telomeres compared to healthy controls, particularly in females. Insignificantly higher global DNA methylation in psoriasis cases compared to the controls indicated marginal clinical importance of this epigenetic test performed in the blood cells' DNA.

## Introduction

The pathogenesis of psoriasis vulgaris involves many metabolic processes that influence the lifespan of lesional skin cells via enhanced proliferation, aberrant differentiation, and down-regulation of pro-apoptotic genes.^1^ Cells present in a psoriasis plaque induce a release of interleukin-1, interleukin-6, tumor necrosis factor-α, interferon-γ and other cytokines that contribute to chronic immunopathological reactions of this disorder.^2^

Apart from skin manifestations, comorbidities, such as psoriatic arthritis, inflammatory bowel disease, cardiovascular diseases, diabetes mellitus, lymphoma or depression are commonly found in psoriatic patients and shorten their lifespan.^3^ In the current theory of aging, genomic instability, telomere attrition, epigenetic alterations, loss of proteostasis, deregulated nutrient-sensing, mitochondrial dysfunction, cellular senescence, stem cell exhaustion, and altered intercellular communication play important roles.^4^, ^5^ The first three mentioned mechanisms associate with DNA changes that might be enhanced in cells and tissues of psoriatic patients.

The process of genomic instability is induced by systemic inflammation and increased oxidative stress present in mild and moderate to severe psoriasis.^6^ In our previous paper, the authors demonstrated elevated serum levels of DNA/RNA oxidized nucleobases, i.e., 8-hydroxy-2′-deoxyguanosine, 8-hydroxyguanosine, and 8-hydroxyguanine in psoriasis, particularly in patients suffering from metabolic syndrome.^7^ Overproduction of reactive oxidative species and DNA damage in their cells might induce accelerated cellular aging and apoptosis.^8^

Telomeres form ends of linear eukaryotic chromosomes, the composition of which in humans consists of 5′-TTAGGG-3′ tandem repeats. A progressive decrease of 50–150 bp of their terminal ends appears in each replication cycle. Critical shortening of telomeres leads to cell senescence, Gap0/Gap1 phase arrest, and apoptosis. Thus, telomeres are considered the molecular clock of cells.^9^ Subsequently, the telomere length in blood cells is considered a promising marker of biological age and aging-related disorders.^10^, ^11^ Tamayo et al. previously reported longer telomere length in rheumatoid arthritis, psoriatic arthritis and ankylosing spondylitis in comparison with healthy subjects.^12^ In contrast, other studies found the opposite or no significant trend in telomere shortening in psoriasis and rheumatologic disorders.^13^, ^14^, ^15^, ^16^

Genome-wide and family-based association studies revealed approximately sixty various genetic loci associated with psoriasis, including several polymorphisms in genes for DNA Methyltransferases (DNMT).^17^, ^18^ These enzymes carry over the epigenetic process of DNA methylation and regulate gene expression and cell proliferation in the body. DNTM1 expression was found significantly elevated in psoriatic peripheral blood mononuclear cells.^19^ In skin, several differentially methylated genes were found to associate with epidermal and keratinocyte differentiation, and cellular regeneration. Hypermethylated promoters in p15, p16, p21, ID4, IFNG, and HLA-C genes affect their expression in psoriasis. Increased p16 and HLA-C promoter methylation, and decreased HLA-DRB1 promoter methylation were significantly associated with the Psoriasis Area Severity Index (PASI score).^20^ On the other side, the authors did not previously find significantly increased methylation of p53, i.e., the gene in which protein product mediates apoptosis in patients with psoriasis.^21^

The aim of this study is to provide more detailed information on oxidation damage, telomere length and methylation status in DNA molecules originating from peripheral blood cells, and to investigate their potential relationships in exacerbated psoriasis vulgaris. In the study, the authors prefer analysis of blood serum and whole-blood DNA to skin nucleic acids. It is expected that results would better correspond with the systemic character of the disease, imbalances in the immune system affecting more tissues and organs than the skin, and processes of biological aging.

## Methods

### Patients

The experimental group consisted of 41 psoriatic patients (21 males and 20 females with a median age of 53 years; range 20–79 years) at the Clinic of Dermal and Venereal Disease, Charles University Hospital in Hradec Kralove. The condition of their disease was assessed using a clinical evaluation of erythema, desquamation, and skin infiltration (PASI score).^22^ According to the PASI score, the psoriasis severity was classified as mild (PASI 0–9), moderate (10–29), or severe form (≥30). The patients did not have any form of psoriasis treatment three months before the study. The subjects with inflammatory diseases (such as infectious diseases, malignancy, and inflammatory rheumatic diseases), pregnancy, and those using nonsteroidal or anti-inflammatory medications were excluded from the study. The control group included 30 healthy blood donors (12 males and 18 females; 53 years; 21–64 years) from the Department of Transfusion Medicine, Charles University Hospital in Hradec Kralove. A power analysis was performed to estimate the required number of patients to include in the study.

All subjects gave their informed consent for inclusion before they participated in the study. The study was conducted in accordance with the Declaration of Helsinki, and the protocol was approved by the Ethics Committee of the Charles University Hospital in Hradec Kralove, Czech Republic (Project identification code PROGRES Q40‒09 and Q40‒10, reference number 201705 I83 P, date 2 May 2017).

For the study purposes, the subjects in experimental and control groups were divided into subgroups depending on the presence/absence of metabolic syndrome. The presence of Metabolic Syndrome (MetS) was evaluated according to the National Cholesterol Education Program Adult Treatment Panel (NCE/ATPIII). MetS diagnosis was declared when three of the following criteria were present: increased waist circumference or abdominal obesity, higher fasting glucose ≥5.6 mmoL/L or known treatment for diabetes, raised the level of triglycerides, reduced level of high-density lipoproteins, and elevated blood pressure.

### Oxidative damage of nucleic acids

Serum from venous blood (BD Vacutainer sampling tubes) was separated by centrifugation and stored at −70 °C until analysis; repeated thawing and freezing were avoided. The level of oxidative damage of nucleic acids was measured via the DNA/RNA Oxidative Damage EIA Kit from Cayman Chemical Company, USA. The values of damage were expressed as the sum of 8-hydroxy-2′-deoxyguanosine released from DNA, 8-hydroxyguanosine from RNA, and 8-hydroxyguanine from either DNA or RNA. Subsequently, the level of DNA/RNA damage was calculated as picograms of all guanine species per milliliter of serum. The detection limit of the kit was 33 pg/mL.

### Length of telomeres

The genomic DNA of the patients and controls was extracted from EDTA venous blood samples according to the manufacturer’s instructions (QIAamp Blood Mini Kit, Germany). The length of telomeres was determined by using the Absolute Human Telomere Length Quantification qPCR Assay Kit (ScienCell, USA) based on direct measurement of the average telomere length via real-time PCR technology (Rotor-Gene 6000, Corbett Research, Australia). According to the manufacturer’s instructions, the obtained data were normalized to the Reference Human Genomic DNA Sample provided in the kit. Finally, the results were expressed as the average telomere length (kilobases, kb) per chromosome end.

### Global DNA methylation

Genomic DNA obtained above (100 ng) was used for the analysis of global DNA methylation. The level of 5-methylcytosine (5-mC) was determined using the 5-mC DNA ELISA Kit (Zymo Research Corporation, USA) according to the manufacturer’s instructions. Denatured, single-stranded DNA samples were coated on good surfaces of a microtiter plate. Anti-5-methylcytosine monoclonal antibody and the HRP-conjugated secondary antibody were added to the wells. After the addition of the HRP developer, photometric detection of 5-mC followed. The 5-mC percentage in DNA samples was quantified according to a standard curve constructed by using commercial standards included in the kit.

### Statistical analysis

Based on the Anderson-Darling test for data distribution, the parametric or nonparametric test was used to ensure test sensitivity (for the differences between the psoriatic patients and the control group). The data were statistically processed with R software version 3.3.2 using the “nortest” and “ggplot2” packages. Associations between all clinical and/or laboratory parameters were evaluated either by Pearson’s or by Spearman’s rank order correlation tests; intergroup differences were assessed using Student’s *t*-test or the Wilcoxon rank-sum test. The present differences were considered statistically significant when the probability level (p) was below the alpha level of 0.05.

## Results

The PASI score median in the patients was 16 (interquartile range 13–22). Five patients had mild form (median PASI 8, range 6–9), 30 patients had moderate form (15, 13–17), and six of them had a severe form of plaque psoriasis (39, 34–43). The weight and BMI values of the patients significantly differed from controls (81 vs. 70 kg, p = 0.009 and 28.3 vs. 24.7 kg/m^2^, p < 0.001, respectively). Twenty-three patients (56%) and five controls (16%) showed signs of metabolic syndrome.

As demonstrated in Table 1, psoriatic patients had a significantly increased median of oxidative damage of DNA/RNA when compared to controls (3758 vs. 2286 pg/mL, p < 0.001). The patients’ values were found significantly higher than the controls in both sexes (males, 3559 vs. 2518 pg/mL, p = 0.033; females, 4184 vs. 1985 pg/mL, p < 0.001). Evaluating oxidation damage separately in the non-smoking subjects, the authors observed significantly higher values of DNA/RNA damage in the non-smoking psoriatic patients (n = 25) than in the non-smoking controls (n = 27): 4228 vs. 2287 pg/mL, p < 0.001. Data on DNA/RNA damage of the patients suffering from MetS did not differ from those without MetS (3559 vs. 3774 pg/mL, p = 0.928). The patients without MetS (n = 18) had significantly higher parameters of DNA/RNA damage than the controls without MetS (n = 25; 3774 vs. 2287 pg/mL, p = 0.001). Statistical analysis did not include control subjects with signs of MetS because of their low number in the study (n = 5).

**Table 1:** Clinical and laboratory data expressed as medians and interquartile ranges.

Variable (unit)	Psoriasis	Controls	p-value
Age (years)			
Females	51 (41–68)	53 (40–58)	ns^c^
Males	60 (44–63)	53 (50–60)	ns
Total	53 (41–67)	53 (44–58)	ns
Weight (kg)			
Females	78 (68–88)	65 (62–71)	0.008
Males	83 (79–100)	81 (71–99)	ns
Total	81 (72–94)	70 (64–83)	0.009
BMI (kg/m^2^)			
Females	28.9 (25.1–32.1)	24.2 (22.8–25.6)	<0.001
Males	28.0 (24.7–30.3)	26.2 (23.7–29.3)	<0.001
Total	28.3 (24.8–30.6)	24.7 (23.2–27.7)	<0.001
DNA/RNA oxidation (pg/mL)			
Females	4184 (2508–5802)	1985 (1559–2451)	<0.001
Males	3559 (2568–4369)	2518 (1905–3091)	0.033
Total	3758 (2563–5269)	2286 (1559–2635)	<0.001
Telomere length (kb)^a^			
Females	3.65 (3.02–4.57)	3.04 (2.47–3.62)	0.020
Males	3.33 (2.98–3.96)	2.95 (2.57–3.50)	ns
Total	3.57 (2.98–4.11)	3.04 (2.51–3.50)	0.011
Global methylation (% 5-mC)^b^			
Females	21.15 (9.65–49.80)	23.35 (2.90–40.10)	ns
Males	39.90 (10.60–57.60)	20.55 (4.60–45.65)	ns
Total	33.20 (10.10–55.60)	23.35 (2.90–40.90)	ns

ns, non-significant.

a Kilobases per chromosome.

b Percentage of 5-methylcytosine in DNA.

c Statistically non-significant result of analysis.

Higher average lengths of telomeres per chromosome were observed in the patients’ blood cells compared to the controls (3.57 vs. 3.04 kb, p = 0.011), particularly in the females (3.65 vs. 3.04 kb, p = 0.020). In the psoriatic males, the telomeres were insignificantly longer compared to the male controls (3.33 vs. 2.95 kb, p = 0.084). The telomere length in the non-smoking psoriatic patients was found significantly longer than in the smoking patients (3.88 vs. 3.24 kb, p = 0.048) and in the non-smoking controls (3.88 vs. 3.03 kb, p = 0.002). The psoriatic patients without MetS revealed longer telomeres (3.84 kb) than the MetS patients (3.38 kb, p = 0.024) and the controls without MetS (3.03 kb, p = 0.001). A negative correlation of the length of telomeres with the age of the control subjects was revealed (Spearman’s rho = -0.420, p = 0.028).

The level of global methylation of the psoriatic DNA (expressed as a percentage of 5-mC DNA) was higher than in the controls, however, the difference did not reach statistical significance (33.20 vs. 23.35%, p = 0.234). Insignificant influences of sex, smoking, BMI, PASI, and MetS on the methylation status were observed. A significant association between methylation status and BMI in the psoriatic patients with MetS was found (Spearman’s rho = 0.417, p = 0.048). No other significant association of the tested variables was apparent.

## Discussion

Nucleic acids contained in cells of psoriatic patients are considered a target of genetic and epigenetic changes possibly qualifying biological processes as apoptosis and premature aging.

The present data showed that the experimental and the control group matched by chronological age distribution. Psoriatic patients had significantly higher weight and BMI than healthy controls. This finding agreed with previous papers reporting increased numbers of psoriatic subjects with metabolic syndrome.^7^, ^23^

Oxidative stress could be caused both by the presence of reactive oxygen species and decreased numbers of antioxidants in the organism.^24^ Former studies manifested that the oxidative changes in nucleic acids of the psoriatic patients positively correlated with the values of the PASI score.^25^ In this study, the authors found a higher oxidative content in the serum DNA/RNA of both the psoriatic females and males compared to the controls. Thus, oxidative stress present in the subjects with exacerbated psoriasis vulgaris of both sexes might contribute to accelerated cellular aging. Further, the authors found a significantly increased content of oxidative DNA/RNA damage in the group of non-MetS patients compared to the non-MetS controls. The oxidative data of the patients with MetS did not differ from those without MetS. The present data suggested that oxidative changes, appearing in nucleic acids as a result of chronic systemic inflammation, are a common part of psoriasis pathophysiology, regardless of the presence or absence of MetS.

Telomeres are repetitive nucleotide elements at the ends of chromosomes that protect them from degradation and genetic information loss. Normal diploid cells lose telomeres in each cell cycle and their actual length may predict the cell lifespan. Telomere lengths in blood DNA accord with those in other tissues and induced telomere shortening are linked to many health issues including aging, aging-related disorders, and cancer.^26^, ^27^ In this study, the authors hypothesized that telomere length in psoriatic blood cells would inversely associate with the level of oxidized DNA/RNA molecules. Preliminarily published studies provided somewhat controversial data on this topic. Blood cell telomere length and urinary levels of oxidized quanine showed a negative correlation in elderly subjects.^28^ T-cells of psoriatic patients showed shorter telomere length.^13^ In contrast, Svenson et al. showed that intense oxidative stress elongates telomeres in granulocytes and naïve T-cells.^11^ A longer telomere length was also reported in leukocytes of subjects with rheumatoid arthritis, psoriatic arthritis, ankylosing spondylitis, systemic scleroderma, and Parkinson’s disease.

In the psoriatic patients, the present study‘s results showed significantly longer telomeres per chromosome than the age-matching controls. Similarly, like in other studies, the length of telomeres in the healthy controls negatively correlated with their age.^12^ This fact corresponded to the general theory of aging.^5^ However, the authors did not observe a similar relationship in the psoriatic subjects. The data revealed sex differences; the lengths of telomeres in the psoriatic females, but not in the males, were significantly higher than those found in the healthy females.

Due to oxidative stress, smoking should cause faster telomere attrition in non-smoking subjects. However, the results of many papers showed contradictory effects of smoking on telomeres. Some of them reported telomere attrition in smokers, other studies found no associations between smoking and the telomere length, while others discovered short telomeres in smokers’ leukocytes, but the telomere attrition rate was found to be slower in the long term.^9^ To minimize the influence of cigarette smoking on the process of telomere attrition, the authors further evaluated the telomere length in non-smoking subjects only. In this subgroup, the authors found significantly longer telomeres in the patients without MetS compared to the appropriate controls; despite that, no association of telomere length with the development of MetS or cardiovascular diseases in psoriasis was previously published.^29^

Based on the present preliminary data, the length of telomeres in blood cells does not seem to correspond with high oxidative damage of DNA molecules present in psoriatic circulation. The reason for the present study‘s findings is not completely clear, and many exogenous and endogenous factors of slower telomere attrition could be considered, including cytokines released from activated immunocytes, enhanced monocytopoietic activity of psoriatic bone marrow, hyperplasia of phagocytes, and others.^30^ Several possible explanations of these findings were suggested by Tamayo et al.: i) Stimulation of telomerase activity in psoriatic leukocytes or primitive hematopoietic progenitors, ii) Dysfunction of shelterin/telosome complex necessary for the capping process, iii) Other cellular or systemic immunological mechanism ensuring correct functions of telomeres, or iv) Epigenetic effect of subtelomeric DNA repeats methylation.^12^

Telomerase catalytic activity adds extra copies of DNA repeat sequences in order to compensate for telomere loss from cell division. Under normal circumstances, this reverse transcriptase activity is restricted mainly to cell types with high proliferation, i.e., germ cells and hemopoietic progenitors. In blood cells, telomerase activity was found in T-cells but not in neutrophils.^9^, ^10^ In rheumatoid arthritis, T-cells exhibited diminished capability of upregulating telomerase activity, despite other authors showing that lymphocytes of the patients show increased telomerase activity.^31^, ^32^ Psoriasis was previously found to induce telomerase activity in T-cells and peripheral blood mononuclear cells, which positively correlated with disease severity.^13^, ^33^ In this way, blood cells might slow telomere shortening, prolong their replicative capacity in blood circulation, and support the immune response of psoriatic patients.

The last part of the present study was focused on the methylation status of psoriatic DNA in peripheral blood cells. The addition of a methyl group to cytosine bases at position 5 of the cytosine ring affects genes linked to immune response, cell cycle regulation, and apoptosis, and it might play an important role in the pathogenesis of psoriasis.^19^ Changes in DNA methylation were found both in the skin and the peripheral blood mononuclear cells. In the skin cells, over a thousand differentially methylated CpG islands were discovered, including hypermethylation of p14 and p16 gene promoters.^34^, ^35^

In the mononuclear cells of psoriatic bone marrow, demethylation of p15, p16, and p21 genes promoters lowering proliferation of hematopoietic cells were observed.^30^ On the other side, FOXP3 gene, which encoded protein is the master regulator of regulatory Treg cell development, was found hypermethylated.^36^ Another study, examining whole blood methylation differences in psoriasis, identified three regions on chromosome-8, and chromosome-6 loci MICA, IRIF1, PSORS1C3, and TNFSF4 as hypermethylated in paternally transmitted disease, while PSORS1C1 was hypomethylated.^37^

Further, significantly increased global methylation of DNA in psoriatic mononuclear cells was announced.^19^ CD4+ and CD8+ T-cells isolated from the whole blood showed no significant differences in methylation or gene expression between affected and unaffected siblings, despite both hypermethylated and hypomethylated states in psoriatics compared to healthy controls reported.^38^ The authors have recently used an epigenetic clock analysis based on sequencing more than 500 age-associated gene loci in psoriatic blood DNA; based on their methylation status, the authors manifested that females suffering from psoriasis were epigenetically older than healthy controls.^3^

In the presented study the authors determined the global DNA methylation by using anti-5-methylcytosine monoclonal antibody and colorimetric detection. The level of methylation in the MetS-patients positively correlated with BMI, nevertheless, no methylation differences between the patients and the control group were found. Formerly, DNA methylation levels determined in skin specimens, but not in peripheral blood mononuclear cells, positively correlated with PASI scores.^19^ Neither of the present results supports any importance of determination of global DNA methylation status in psoriasis blood DNA. Although the 5-mC DNA median, determined in the patients (33.20%), was apparently higher than in the controls (23.35%), the statistical significance of the difference was disabled by a wide fluctuation of 5-mC DNA values in both groups (10.10–55.60% in the patients vs 2.90–40.90% in the controls, see Table 1), and by a relatively low number of cases reported in the study. It is possible that thousands of hypo- and hypermethylated sites in the genes pathophysiologically associated with psoriasis could hide individual methylation changes that appear in psoriatic blood cells' DNA.

Summarizing the authors ‘findings, this study showed the consequences of oxidative DNA/RNA damage and modifications of telomeres with psoriasis pathophysiology. Both processes can significantly influence the proliferation activities of cells. Increased levels of oxidized nucleic acids in psoriatic serum reflect the presence of chronic systemic inflammation, mainly in patients with a higher weight and BMI, and with MetS. Elevated levels of 8-hydroxy-2′-deoxyguanosine, 8-hydroxyguanosine, and 8-hydroxyguanine seem to be promising serum biomarkers of oxidative damage in nucleic acids and accelerated cellular aging. Their levels could be therapeutically influenced by antioxidant treatment.

In psoriatic blood cells, the authors observed longer telomeres than in healthy controls. However, the pathophysiological roles of telomeric changes in psoriasis are currently unclear. Their biochemical pathways might lead to induced telomerase activity, lower numbers of cells underlying apoptosis, and stronger immune response. Several substances like thapsigargin were reported to experimentally modify the length of telomeres and telomerase catalytic activity in keratinocytes.^39^

The present study shows, however, several limitations. First, it had a cross-sectional design and contained a limited number of included participants, particularly for reliable subgroup analyses. Another limitation of the study was the Caucasian origin of the participants. As ethnicity, climate, geography and other environmental factors could influence the results of the parameters determined, broader multicenter studies should verify the preliminary results. Third, the authors did not directly determine telomerase activity in serum or blood cells. Finally, further studies with a closer look at oxidation, telomere, telomerase activity and methylation processes in various skin locations, individual cellular lineages, and specific genes could help us to better understand these processes in the pathogenesis of psoriasis vulgaris.

## Conclusion

The study demonstrated increased levels of oxidized DNA/RNA molecules in the serum of patients with exacerbated psoriasis vulgaris. The results were minimally influenced by sex, the presence of metabolic syndrome or cigarette smoking. In the psoriatic blood cells' DNA, the authors observed longer telomeres compared to the healthy controls, particularly in the females. Insignificantly higher global DNA methylation in psoriasis cases compared to the controls indicated marginal clinical importance of this epigenetic test performed in the blood cells' DNA.

## Financial support

This research was funded by the projects COOPERATIO UK and SVV-260543/2020 of Charles University, Faculty of Medicine in Hradec Kralove, Czech Republic.

## Authors' contributions

Martin Beranek: Critical literature review; data collection, analysis, and interpretation; preparation and writing of the manuscript.

Pavel Borsky: Data collection, analysis, and interpretation; effective participation in research orientation; statistical analysis; study conception and planning.

Zdenek Fiala: Approval of the final version of the manuscript; manuscript critical review.

Ctirad Andrys: Data collection, analysis, and interpretation; study conception and planning.

Kvetoslava Hamakova: Effective participation in research orientation; study conception and planning.

Marcela Chmelarova: Data collection, analysis, and interpretation.

Helena Kovarikova: Data collection, analysis, and interpretation.

Adam Karas: Data collection, analysis, and interpretation.

Jan Kremlacek: Statistical analysis.

Vladimir Palicka: Approval of the final version of the manuscript; manuscript critical review.

Lenka Borska: Effective participation in research orientation; manuscript critical review; study conception and planning.

## Conflicts of interest

None declared.

## References

[1] Eding C.B., Enerbäck C. (2017). Involved and uninvolved psoriatic keratinocytes display a resistance to apoptosis that may contribute to epidermal thickness. Acta Derm Venereol..

[2] Owczarczyk-Saczonek A., Czerwinska J., Placek W. (2018). The role of regulatory T cells and anti-inflammatory cytokines in psoriasis. Acta Dermatovenerol Alp Pannonica Adriat..

[3] Borsky P., Chmelarova M., Fiala Z., Hamakova K., Palicka V., Krejsek J. (2021). Aging in psoriasis vulgaris: female patients are epigenetically older than healthy controls. Immun Ageing..

[4] Moskalev A.A., Shaposhnikov M.V., Plyusnina E.N., Zhavoronkov A., Budovsky A., Yanai H. (2013). The role of DNA damagee and repair in aging through the prism of Koch-like criteria. Ageing Res Rev..

[5] Lopez-Otin C., Blasco M.A., Partridge L., Serrano M., Kroemer G. (2013). The hallmarks of aging. Cell..

[6] Basavaraj K.H., Vasu Devaraju P., Rao K.S. (2013). Studies on serum 8-hydroxy guanosine (8-OHdG) as reliable biomarker for psoriasis. J Eur Acad Dermatol Venereol..

[7] Borska L., Kremlacek J., Andrys C., Krejsek J., Hamakova K., Borsky P. (2017). Systemic inflammation, oxidative damage to nucleic acids, and metabolic syndrome in the pathogenesis of psoriasis. Int J Mol Sci..

[8] Borska L., Andrys C., Krejsek J., Palicka V., Vorisek V., Hamakova K. (2016). Influence of dermal exposure to ultraviolet radiation and coal tar (polycyclic aromatic hydrocarbons) on the skin aging process. J Dermatol Sci..

[9] Kordinas V., Ioannidis A., Chatzipanagiotou S. (2016). The telomere/telomerase system in chronic inflammatory diseases. Cause or effect?. Genes (Basel)..

[10] Robertson J.D., Gale R.E., Wynn R.F., Dougal M., Linch D.C., Testa N.G. (2000). Dynamics of telomere shortening in neutrophils and T lymphocytes during ageing and the relationship to skewed X chromosome inactivation patterns. Br J Haematol..

[11] Svenson U., Nordfjäll K., Baird D., Roger L., Osterman P., Hellenius M.L. (2011). Blood cell telomere length is a dynamic feature. PLoS One..

[12] Tamayo M., Mosquera A., Rego J.I., Fernández-Sueiro J.L., Blanco F.J., Fernández J.L. (2010). Differing patterns of peripheral blood leukocyte telomere length in rheumatologic diseases. Mutat Res..

[13] Wu K., Higashi N., Hansen E.R., Lund M., Bang K., Thestrup-Pedersen K. (2000). Telomerase activity is increased, and telomere length shortened in T cells from blood of patients with atopic dermatitis and psoriasis. J Immunol..

[14] Schonland S.O., Lopez C., Widmann T., Zimmer J., Bryl E., Goronzy J.J. (2003). Premature telomeric loss in rheumatoid arthritis is genetically determined and involves both myeloid and lymphoid cell lineages. Proc Natl Acad Sci USA..

[15] Yudoh K., Nguyen T., Nakamura H., Hongo-Masuko K., Kato T., Nishioka K. (2005). Potential involvement of oxidative stress in cartilage senescence and development of osteoarthritis: oxidative stress induces chondrocyte telomere instability and downregulation of chondrocyte function. Arthritis Res Ther..

[16] Wu C.H., Hsieh S.C., Li K.J., Lu M.C., Yu C.L. (2007). Premature telomere shortening in polymorphonuclear neutrophils from patients with systemic lupus erythematosus is related to the lupus disease activity. Lupus..

[17] Chandra A., Ray A., Senapati S., Chatterjee R. (2015). Genetic and epigenetic basis of psoriasis pathogenesis. Mol Immunol..

[18] Tsoi L.C., Stuart P.E., Tian C., Gudjonsson J.E., Das S., Zawistowski M. (2017). Large scale meta-analysis characterizes genetic architecture for common psoriasis associated variants. Nat Commun..

[19] Zhang P., Su Y., Chen H., Zhao M., Lu Q. (2010). Abnormal DNA methylation in skin lesions and PBMCs of patients with psoriasis vulgaris. J Dermatol Sci..

[20] Whyte J.M., Ellis J.J., Brown M.A., Kenna T.J. (2019). Best practices in DNA methylation: lessons from inflammatory bowel disease, psoriasis and ankylosing spondylitis. Arthritis Res Ther..

[21] Borsky P., Chmelarova M., Fiala Z., Palicka V., Beranek M., Kremlacek J. (2020). Variation of selected genotoxic and epigenetic markers due to therapeutic exposure to PAHs and ultraviolet radiation. Bratisl Lek Listy..

[22] Salihbegovic E.M., Hadzigrahic N., Cickusic A.J. (2015). Psoriasis and metabolic syndrome. Med Arch..

[23] Milcic D., Jankovic S., Vesic S., Milinkovic M., Marinkovic J., Cirkovic A. (2017). Prevalence of metabolic syndrome in patients with psoriasis: A hospital-based cross-sectional study. An Bras Dermatol..

[24] Barygina V., Becatti M., Lotti T., Taddei N., Fiorillo C. (2016). Low dose cytokines reduce oxidative stress in primary lesional fibroblasts obtained from psoriatic patients. J Dermatol Sci..

[25] Holmannova D., Borska L., Andrys C., Borsky P., Kremlacek J., Hamakova K. (2020). The impact of psoriasis and metabolic syndrome on the systemic inflammation and oxidative damage to nucleic acids. J Immunol Res..

[26] Cawton R.M., Smith K.R., O’Brien E., Silvatchenko A., Kerber R.A. (2003). Association between telomere length in blood and mortality in people aged 60 years or older. Lancet..

[27] Njajou O.T., Cawthon R.M., Damcott C.M., Wu S.H., Ott S., Garant M.J. (2007). Telomere length is paternally inherited and is associated with parental lifespan. Proc Natl Acad Sci USA..

[28] Tosevska A., Franzke B., Hofmann M., Vierheilig I., Schober-Halper B., Oesen S. (2016). Circulating cell-free DNA, telomere length and bilirubin in the Vienna Active Ageing Study: exploratory analysis of a randomized, controlled trial. Sci Rep..

[29] Coussens E., Grine L., Bostoen J., Mielants H., Lambert J. (2016). Analysis of telomere length as predictive marker in psoriasis for comorbidities. Exp Dermatol..

[30] Zhang K., Zhang R., Li X., Yin G., Niu X., Hou R. (2007). The mRNA expression and promoter methylation status of the p16 gene in colony-forming cells with high proliferative potential in patients with psoriasis. Clin Exp Dermatol..

[31] Fujii H., Shao L., Colmegna I., Goronzy J.J., Weyand C.M. (2009). Telomerase insufficiency in rheumatoid arthritis. Proc Natl Acad Sci USA..

[32] Dehbi A.Z., Radstake T.R., Broen J.C. (2013). Accelerated telomere shortening in rheumatic diseases: Cause or consequence?. Expert Rev Clin Immunol..

[33] Liu X., Lin J., Wang L., Zhang Z., Hou S., Yang G. (2008). Telomerase activity in peripheral blood mononuclear cells of psoriatic patients correlates with disease severity. Br J Dermatol..

[34] Roberson E.D., Liu Y., Ryan C., Joyce C.E., Duan S., Cao L. (2012). A subset of methylated CpG sites differentiate psoriatic from normal skin. J Invest Dermatol..

[35] Chen M., Chen Z.Q., Cui P.G., Yao X., Li Y.M., Li A.S. (2008). The methylation pattern of p16INK4a gene promoter in psoriatic epidermis and its clinical significance. Br J Dermatol..

[36] Ngalamika O., Liang G., Zhao M., Yu X., Yang Y., Yin H. (2015). Peripheral whole blood FOXP3 TSDR methylation: a potential marker in severity assessment of autoimmune diseases and chronic infections. Immunol Invest..

[37] Pollock R.A., Abji F., Gladman D.D. (2017). Epigenetics of psoriatic disease: A systematic review and critical appraisal. J Autoimmun..

[38] Mervis J.S., McGee J.S. (2020). DNA methylation and inflammatory skin diseases. Arch Dermatol Res..

[39] Rosenberger S., Thorey I.S., Werner S., Boukamp P. (2007). A novel regulator of telomerase. S100A8 mediates differentiation-dependent and calcium-induced inhibition of telomerase activity in the human epidermal keratinocyte line HaCaT. J Biol Chem..

